# Exploring the causality between ankylosing spondylitis and atrial fibrillation: A two-sample Mendelian randomization study

**DOI:** 10.3389/fgene.2022.951893

**Published:** 2022-11-16

**Authors:** Shuhong Chen, Xiqing Luo, Jiaoshi Zhao, Zhenguo Liang, Jieruo Gu

**Affiliations:** ^1^ Department of Rheumatology, The Third Affiliated Hospital of Sun Yat-sen University, Guangzhou, China; ^2^ The Clinical Medical Research Center for Immune Diseases of Guangdong Province, Guangzhou, China

**Keywords:** atrial fibrillation, Mendelian randomization, genome-wide association study, ankylosing spondylitis, genetic correlation

## Abstract

**Objective:** To study whether ankylosing spondylitis (AS) has a causal effect on the risk of atrial fibrillation (AF) using two-sample Mendelian randomization (MR) analysis.

**Methods:** Single nucleotide polymorphisms (SNPs) were selected as independent instrumental variables (IVs) from a GWAS study of AS. Summary data from a large-scale GWAS meta-analysis of AF was utilized as the outcome dataset. Inverse-variance weighted (IVW) model was used for the primary analysis. Multiple sensitivity and heterogeneity tests were conducted to confirm the robustness of the results.

**Results:** In total, 18 SNPs were identified as IVs for MR analysis. Five MR methods consistently found that ankylosing spondylitis was not causally associated with atrial fibrillation (IVW: OR = 0.983 (0.894, 1.080), *p* = 0.718; MR-Egger: OR = 1.190 (0.973, 1.456), *p* = 0.109; Simple mode: OR = 0.888 (0.718, 1.098), *p* = 0.287; Weighted mode: OR = 0.989 (0.854, 1.147), *p* = 0.890; Weight median: OR = 0.963 (0.852, 1.088), *p* = 0.545). Leave-one-out analysis supported the stability of MR results. Both the MR-Egger intercept and MR-PRESSO method revealed the absence of horizontal pleiotropy.

**Conclusion:** The two-sample MR analysis did not support a causal relationship between AS and the risk of AF.

## Introduction

Atrial fibrillation (AF) is the most common arrhythmia characterized by rapid and irregular atrial activation (Lip et al., 2012). AF is associated with poor life quality and increasing morbidity and mortality (Staerk et al., 2017; Virani et al., 2021), resulting in a heavy socioeconomic burden (Chugh et al., 2014). AF is a multifactorial complex disease involving modifiable risk factors (e.g., smoking, obesity, and hypertension) and unmodifiable risk factors (e.g., genetics, age, and male) (Young et al., 2022). Despite incomplete understanding of the pathogenesis, inflammation is a well-established core process contributing to not only atrium electronic and structural remodeling but also thrombogenesis, increasing the vulnerability to AF. In turn, AF exacerbates local inflammation in the atrium, resulting in the recurrence of AF and ultimately the vicious circle (Hu et al., 2015). Clinical data has suggested an increase in serum IL-6 and C-reactive protein (CRP) level of patients with AF compared to health control (Guo et al., 2012). Substantial evidence has suggested that patients with chronic inflammatory conditions are prone to AF, underscoring the need for early management in these patients (Gawałko et al., 2020; Khan et al., 2021).

As an inflammatory arthritis, ankylosing spondylitis (AS) is characterized by inflammation and bone hyperplasia at axial skeletons, leading to immobility and even lifetime disability (Braun and Sieper, 2007). Heart involvement occurs in 10%–30% patients with AS, including aortitis, valvular heart diseases, and cardiac conduction abnormalities (El Maghraoui, 2011). Multiple studies have reported higher CV morbidity and mortality in AS population compared to the general population. Given the intertwined relationship between CV morbidity and mortality and AF, some researchers have assessed the risk of AF in patients with AS (Kim and Choi, 2021). A recent meta-analysis has included three relevant cohort studies and observed a positive association between AS and 85% higher risk of AF with little heterogeneity (Morovatdar et al., 2021). However, it is difficult to determine whether the causation underlies the observational association. In addition, the etiology of increased AF risk in patients with AS remains unknown. Although systemic inflammation is one of putative mechanisms, the development of AF might be attributed to higher prevalence of traditional CV risk factors and a high frequency of NSAID usage in AS patients (Atzeni et al., 2020). Given overlapping genetic architecture between AS and CRP level, whether such genetic components related to inflammation are implicated in the observational association remains to be explored (International Genetics of Ankylosing Spondylitis Consortium et al., 2013). Further research is warranted to decipher the relationship between AS and incident atrial fibrillation.

Mendelian randomization (MR) analysis is an epidemiological method that uses genetic variants as instrument variables (IVs) to make causal inferences, avoiding the biases of observational studies (Didelez and Sheehan, 2007; Davey Smith and Hemani, 2014). Leveraging the random allocation of variants during the process of gametogenesis, MR is able to mimic randomize controlled trials and explore some intractable questions of causality due to high cost or ethical issues. With the boom of genome-wide association studies (GWAS) in the past decade, MR has become widespread for the identification of risk factors and biomarkers for diseases/traits. In this study, based on large-scale GWAS datasets, we aimed to explore the causal effect between AS and AF in a two-sample Mendelian randomization analysis framework. Additionally, linkage disequilibrium regression score (LDSC) analysis was conducted to investigate cross-trait genetic correlation between AS and AF.

## Materials and methods

### Data sources and study design

The exposure dataset was obtained from the IGAS (International Genetics of Ankylosing Spondylitis) Consortium for ankylosing spondylitis (International Genetics of Ankylosing Spondylitis Consortium et al., 2013). The diagnosis of AS was based on the modified New York criteria. It included 10,619 AS cases and 15,145 controls of multiple ancestries from 11 countries. Considering the bias from population stratification, the analysis exclusively included the population of European ancestry. Hence, our MR analysis ultimately included only the European cohort from the IGAS GWAS dataset for AS (9,069 cases and 13,578 controls). The outcome dataset was derived from the large-scale GWAS meta-analysis for atrial fibrillation (Nielsen et al., 2018). It consisted of 60,620 AF cases and 970,216 controls from six biobanks, including the Nord-Trøndelag Health Study (HUNT), deCODE, the Michigan Genomics Initiative (MGI), DiscovEHR, UK Biobank, and the AFGen Consortium. The AF cases were selected from biobanks using electronic health records and International Classification of Diseases (ICD) codes (ICD-9 and ICD-10). There was no clear evidence for sample overlapping between the exposure dataset and the outcome dataset. Details of the datasets are listed in Table 1.

**TABLE 1:** Data sources of summary datasets.

	Trait	Sample size	nSNP	Year	Ancestry	PMID
Exposure	Ankylosing spondylitis	22,647	99,962	2013	European	23749187
Outcome	Atrial fibrillation	1,030,836	33,519,037	2018	European	30061737

**FIGURE 1:**
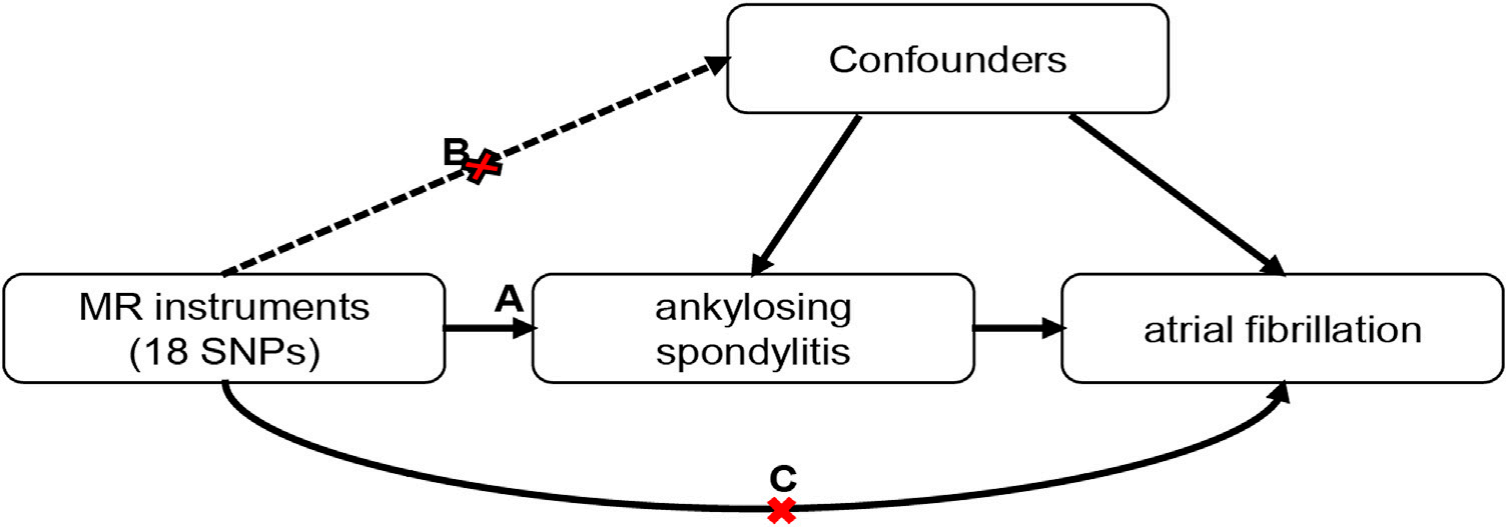
Schematic of the Mendelian randomization framework. Three core assumptions were as follows: **(A)** the SNPs should be strongly associated with ankylosing spondylitis; **(B)** the SNPs should not be related to confounders; **(C)** the SNPs should not be directly associated with atrial fibrillation.

A two-sample MR approach was performed to investigate the potential causality between AS and AF. The MR analysis followed strictly the STROBE-MR Statement (Skrivankova et al., 2021). The study design is shown in Figure 1. The MR method should fulfill three assumptions: First, the genetic variants should be associated with AS; Second, the genetic variants should not be related to any confounders. Third, the genetic variants exert effects on AF only *via* AS.

### Selection of instrument variables

SNPs at the genome-wide significance level (*p* < 5 × 10^−8^) were extracted from the IGAS GWAS summary data. To eliminate biases from linkage disequilibrium (LD), we only fetched independent SNPs with *r* ^2^ < 0.001 within the distance of 10,000 kb (G et al., 2018). We searched the PhenoScanner website (http://www.phenoscanner.medschl.cam.ac.uk) for each SNP to evaluate any prior reported association with AF or its known risk factors (*p* < 1 × 10^−5^) (Kamat et al., 2019). Hence, we cut out seven variants related to blood pressure, lipid parameters, and diabetes (Supplementary Table S1). We removed the SNP rs130075 due to its missing effect size and selected 18 SNPs as IVs. All the SNPs were available in the summary dataset of AF. To avoid weak instrumental variable bias, we calculated the F statistic of each IV-SNP using the following equation:

$$F=\frac{R^{2}\left(N-k-1\right)}{\left(1-R^{2}\right)}$$

where *R* ^2^ is the genetic variance explained by each SNP, *N* is the sample size of the exposure dataset and *k* is the number of IVs. IVs with an *F*-statistic below 10 were considered as weak instrument and excluded. In addition, we calculated the statistical power with an alpha of 0.05 using an interactive analysis tool (http://cnsgenomics.com/shiny/mRnd/) (Brion et al., 2013; Davies et al., 2015).

### Two sample Mendelian randomization

The primary MR analysis was the inverse-variance weighted (IVW) method, the most accurate MR method based on the assumption that all SNPs are valid instrument variables. For each SNP, the causal effect was calculated as the Wald ratio of SNP-exposure effect to SNP-outcome effect. Then, we used the fixed-effect inverse variance weighting method to obtain the total effect value by summarizing causal estimates across all SNPs (Burgess et al., 2013).

### Sensitivity and heterogeneity analysis

Several sensitivity analyses were conducted to test the robustness of causal effects (Burgess et al., 2017; Zhang and Ghosh, 2021). First, the size and direction of effect estimates were compared across 5 MR methods: IVW, MR-Egger (Burgess and Thompson, 2017), weighted median (WM^1^) (Bowden et al., 2016), weighted mode (WM^2^), and simple mode (SM) (Hartwig et al., 2017). Due to different assumptions of these methods, consistency of effect estimates is the most robust evidence for causality. Second, leave-one-out analysis can evaluate whether the results are markedly affected by individual SNPs. In addition, the heterogeneity was qualified by the Cochran’s Q statistic or the *I* ^2^ statistic. The Q statistic with a *p*-value < 0.05 or *I* ^2^ > 75% is indicative of the presence of heterogeneity (Burgess et al., 2017). A random-effect IVW model will be adopted if any heterogeneity exists. For pleiotropy tests, either MR-Egger regression (Bowden et al., 2015) with an intercept near-zero or MR pleiotropy residual sum and outlier (MR-PRESSO) method with *p*-value < 0.05 supports the presence of horizontal pleiotropy (Hemani et al., 2018b; Verbanck et al., 2018).

### Genetic correlation analysis

Genetic correlation analysis was performed to compute *R*_g_ and *p*-value by using the ldsc software to evaluate the shared genetic contribution of AS and AF (Bulik-Sullivan et al., 2015).

### Statistical analysis

The R packages “MendelianRandomization” (Yavorska and Burgess, 2017) and “TwoSampleMR” (Hemani et al., 2018a) were used for the MR and sensitivity analyses. The R package “MRPRESSO” was used for the MR-PRESSO test (Verbanck et al., 2018). All MR analyses were performed in the R platform (version: 4.1.1). The software “ldsc” were used for genetic correlation analysis (Bulik-Sullivan et al., 2015). The two-sided *p* < 0.05 was set as the significant threshold.

### Ethics statement

In this MR analysis, we only used the summary-level data of public available GWAS studies. All the GWAS studies have received ethical approval from the corresponding institutional review boards. Thus, no more ethical committee approval was required for this study. The IGAS GWAS study was approved by the corresponding research ethics boards at each study center. The UK Biobank was approved by the North West Multicenter Research Ethics Committee, the National Information Governance Board for Health and Social Care in England and Wales, and the Community Health Index Advisory Group in Scotland.

## Results

### Identification of instrument variables

In total, 18 SNPs were selected as IVs for AS. Table 2 describes the detailed information of 18 SNPs. The *F*-statistics of SNPs ranged from 30 to 360, indicating the absence of weak IV bias. These SNPs explained 17.0% of genetic variance for AS in total. Under the sample size (60,620 AF cases and 970,216 controls), the statistical power reached 100% to detect the estimated causal effect size of AS on AF (RR = 1.85) at an alpha of 0.05 (Morovatdar et al., 2021).

**TABLE 2:** The information of 18 IV SNPs in MR analysis.

SNP	CHR	Position	EA/NEA	beta	SE	*p* val	F-stat
rs11209026	1	67705958	A/G	−0.104	0.010	1.94E-27	118
rs41299637	1	200877850	G/T	−0.039	0.005	1.81E-15	63
rs6600247	1	25305114	C/T	0.033	0.004	2.58E-15	63
rs4672505	2	62560332	G/A	−0.050	0.004	5.14E-47	207
rs4676410	2	241563739	A/G	0.028	0.005	9.90E-09	33
rs12615545	2	182048452	C/T	0.025	0.004	1.03E-09	37
rs27529	5	96126308	G/A	−0.062	0.004	3.28E-47	208
rs6556416	5	158818745	C/A	0.025	0.005	4.22E-08	30
rs1041926	6	28426296	A/G	−0.074	0.012	1.55E-10	41
rs2517655	6	30121048	T/C	0.088	0.005	3.47E-80	360
rs11190133	10	101278725	T/C	−0.034	0.004	4.84E-14	57
rs1250550	10	81060317	A/C	−0.026	0.004	1.46E-09	37
rs1860545	12	6446777	A/G	−0.027	0.004	2.78E-10	40
rs11624293	14	88488821	C/T	0.043	0.007	1.49E-10	41
rs2531875	17	26148167	T/G	−0.027	0.004	1.22E-10	41
rs35164067	19	10525181	A/G	−0.031	0.005	3.43E-10	39
rs2836883	21	40466744	A/G	−0.040	0.005	6.46E-17	70
rs743479	21	45611950	T/C	−0.023	0.004	2.03E-08	31

*SNP, single nucleotide polymorphism; CHR, chromosome; EA, effect allele; NEA, non-effect allele; SE, standard effect.

### Mendelian randomization analysis

**FIGURE 2:**
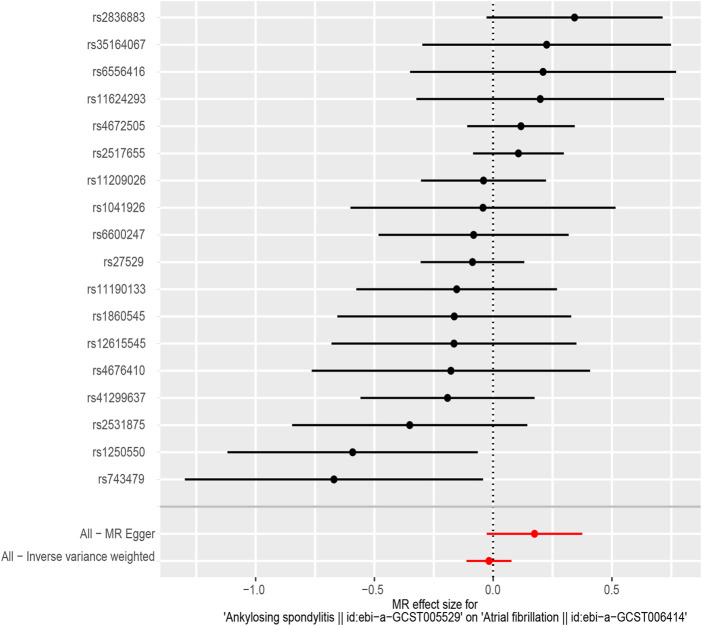
The forest plot of Mendelian randomization analysis for 18 SNPs.

**FIGURE 3:**
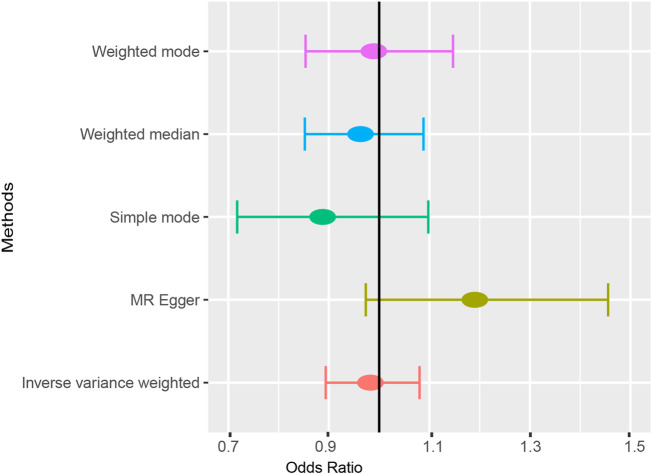
The forest plot of five Mendelian randomization methods.

According to the results of the IVW method, there was no MR association between genetically determined ankylosing spondylitis and atrial fibrillation [OR = 0.983 (0.894, 1.080), *p* = 0.718]. As shown in Table 3, similar results were obtained from four other methods (MR-Egger: OR = 1.190 [0.973, 1.456], *p* = 0.109; WM^1^: OR = 0.963 [0.852, 1.088], *p* = 0.545; WM^2^: OR = 0.989 [0.854, 1.147], *p* = 0.890; SM: OR = 0.888 (0.718, 1.098), *p* = 0.287). It strongly supported no evidence of causality between AS and AF (Figures 2, 3).

**TABLE 3:** Causal effect estimates of AS on AF from 5 MR methods.

Method	nSNP	beta	SE	OR	95% CI	*p*-value
MR-Egger	18	0.174	0.103	1.190	0.973, 1.456	0.109
WM1	18	−0.038	0.062	0.963	0.852, 1.088	0.545
IVW	18	−0.017	0.048	0.983	0.894, 1.080	0.718
SM	18	−0.119	0.108	0.888	0.718, 1.098	0.287
WM2	18	−0.011	0.075	0.989	0.854, 1.147	0.890

*WM1, weighted median; SM, simple mode; WM2, weighted mode; OR, odds ratio; CI, confidence interval.

### Heterogeneity and sensitivity tests

**FIGURE 4:**
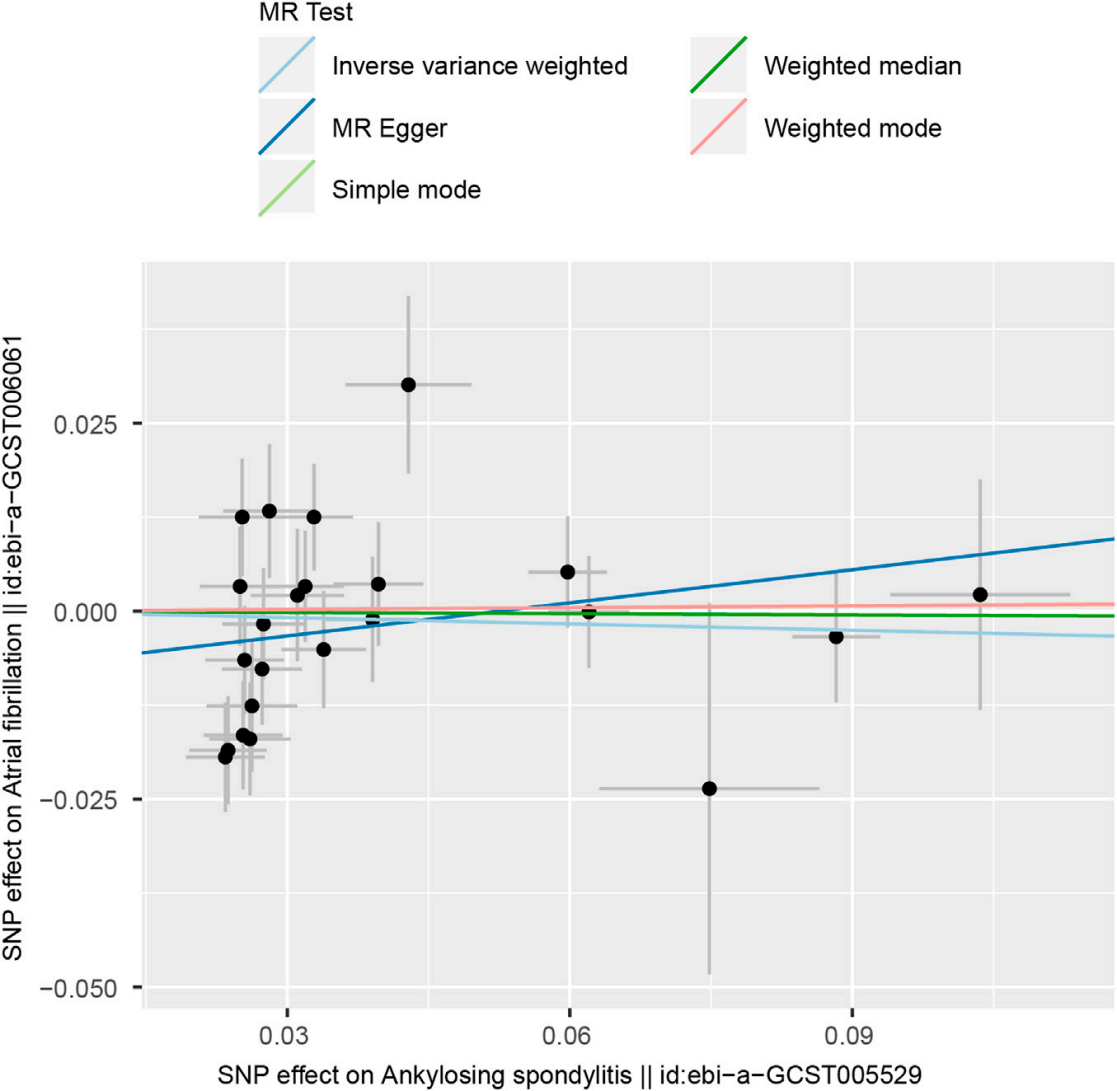
The scatter plot of five Mendelian randomization methods.

**FIGURE 5:**
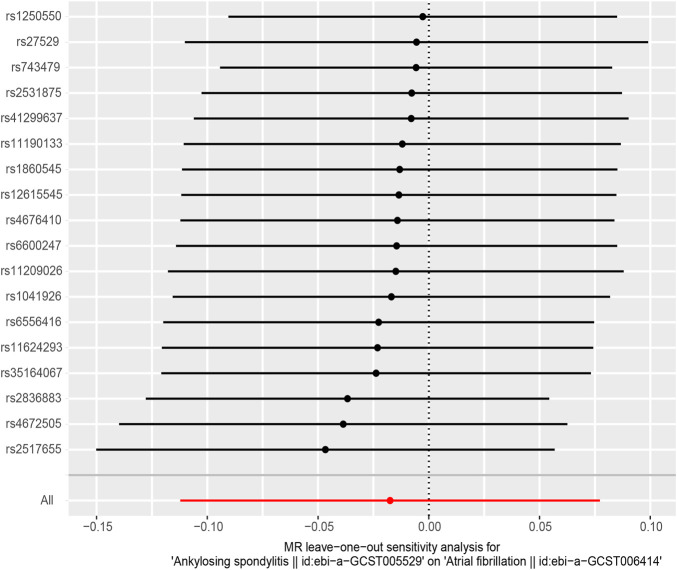
The forest plot of leave-one-out analysis.

**FIGURE 6:**
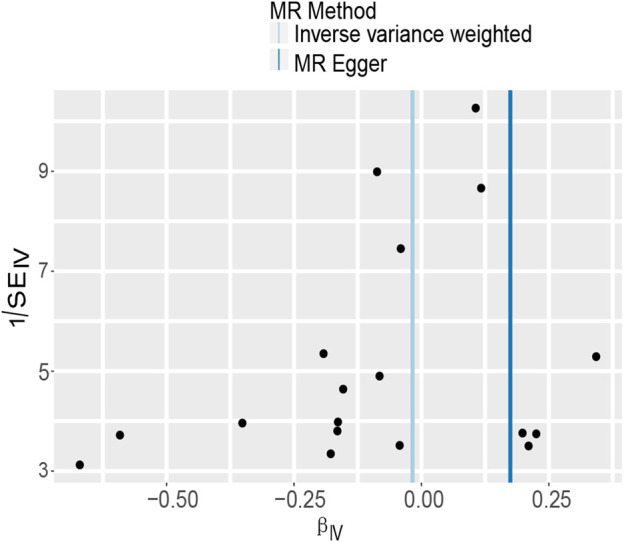
The funnel plot of Mendelian randomization analysis.

In MR-Egger regression analysis, the intercept was close to zero (Figure 4), suggesting no horizontal pleiotropy which was further validated by the MR-PRESSO test (*p* = 0.185). Heterogeneity tests suggested low heterogeneity across the SNPs (*I* ^2^ = 0.102; IVW: *Q* = 21.93, *p* = 0.187; MR-Egger: *Q* = 17.30, *p* = 0.366). Leave-one-out analysis found that the results were not markedly affected by any single SNP (Figure 5). No outlier SNP was detected by the MR-PRESSO test. The funnel plot provided no evidence for horizontal pleiotropy (Figure 6).

### Genetic correlation between ankylosing spondylitis and atrial fibrillation

Based on the hypothesis that the epidemiological association between two diseases may root from common susceptible genetic variants, we evaluated the genetic correlation between AS and AF by LDSC analysis. However, we did not observe a significant genetic correlation between these two diseases (*R*_g_ = 0.1179, *p* = 0.2115, Table 4). It has indicated that the higher incidence of AF in patients with AS is not caused by shared genetic contributions.

**TABLE 4:** The genetic correlation results of LDSC analysis.

Phenotype_1_	Phenotype_2_	R_g_	R_g__SE	R_g__Z	*p*
Ankylosing spondylitis	Atrial fibrillation	0.1179	0.0943	1.2496	0.2115

*Rg, genetic correlation estimate; Rg_SE, standard error of genetic correlation estimate; Rg_Z, z-score of genetic correlation estimate.

## Discussion

This is the first study to explore the causal relationship between AS and AF by MR analysis. MR estimates from five methods (IVW, WM^1^, SM, WM^2^, and MR-Egger) suggested no evidence of causality between AS and AF. The heterogeneity and sensitivity tests verified the robustness of our results. In LDSC analysis, no genetic correlation existed between AS and AF. This study shed light on the relationship between AS and the risk of incident AF from a genetic perspective.

Cardiovascular involvement is common in ankylosing spondylitis featuring aortitis, valvular diseases, and conduction disturbances (Gensler, 2015). Recently, researchers have shifted their focus to atrial arrhythmia in AS patients by performing 12-lead electrocardiography (ECG) test and 24 h Holter monitoring and yielded limited data. Cumulative evidence from observational studies has suggested that the abnormalities of P-wave morphology in ECG can predict new-onset atrial fibrillation (Yoshizawa et al., 2014; Gulsen et al., 2020). Yildirir et al. (2002) reported no difference in signal-averaged P-wave duration between AS patients and controls whereas two other studies have observed prolonged P-wave dispersion and maximum P-wave duration, which conferred a higher risk of AF (Acar et al., 2009; Aksoy et al., 2016). In most studies, Holter examination has found that supraventricular arrhythmias (AF included) occurred more frequently in AS patients (Ho et al., 2012; Aksoy et al., 2016) while few studies have reported no difference (Kazmierczak et al., 2007). However, these studies mentioned above were cross-sectional and small-scale. The outcome variables in these studies were mostly ECG and Holter parameters rather than the diagnosis of AF. To fill these gaps, two nationwide prospective studies have followed up AS patients for several years to evaluate the incidence of AF (Bengtsson et al., 2019; Moon et al., 2019). Both studies have demonstrated that AS patients were at higher risk of new-onset AF. Although both studies have attempted to adjust some confounding factors such as age, gender, and classical cardiometabolic risk factors, the flaws still remain in the observational study designs viz selection bias and reverse causation. It is difficult to distinguish the causality from the association mediated by confounders.

This MR study is the first of its kind to investigate the causal relationship between AS and AF, diminishing biases in observational studies. The results did not support the causality from AS to AF. Furthermore, the observational association cannot be explained by their shared genetic contributions. However, there are a few possible explanations for the increased risk of AF in patients with AS.

First, this non-causal association may be confounded by traditional risk factors for AF. Smoking (Zhu et al., 2016) and low physical activity (Khurshid et al., 2021) are two well-established traditional risk factors for AF. In fact, the prevalence of smoking and low physical activity due to back pain in AS cohort is higher than the general population. Smoking and low physical activity may predispose patients with AS to new-onset atrial fibrillation. Alternatively, patients with AS are associated with high prevalence of comorbidities like hypertension, diabetes, valve diseases, and heart failure, which are known risk factors for AF (Toussirot, 2021).

Second, systemic inflammation from AS could play a key role in the initiation and maintenance of AF (Hu et al., 2015). On the one hand, proinflammatory macrophages and lymphocytes migrate and infiltrate into atrial tissues (Fontes et al., 2005; Chen et al., 2008), leading to local inflammation, structural and electronic remodeling (De Jong et al., 2011; Jalife and Kaur, 2015). On the other hand, systemic inflammation is contributed to the increase of AF risk in patients with autoimmune diseases (Ciconte et al., 2018). Several inflammatory biomarkers are used to predict the risk and prognosis of AF (Hijazi et al., 2016; Jabati et al., 2018). Furthermore, the causal relationship between IL-6 and AF has been supported by MR analysis, but not for CRP and AF (Yuan et al., 2020; Li et al., 2022). Another line of evidence comes from the antiarrhythmic effects of anti-inflammatory agents like anti-TNF agents and colchicine (Saljic et al., 2022). Interestingly, the Korean nationwide study afore has suggested that the use of anti-TNF agents was a risk factor for AF, which needs to be further explored (Moon et al., 2019). In summary, systemic inflammation in patients with AS may be a mediator of new-onset AF.

Third, non-steroidal anti-inflammatory drugs (NSAIDs) are the first-line therapy for AS while they carry an increased risk for AF (Chokesuwattanaskul et al., 2020). NSAIDs render patients more prone to arrhythmias by reducing endogenous antiarrhythmic agent prostacyclin (Pepine and Gurbel, 2017) and enhancing the renin angiotensin system (Asghar et al., 2017). Furthermore, NSAIDs have increased the risk of hypertension, heart failure and myocardial infarction, all of which are risk factors of AF (Braun et al., 2020). Thus, patients with AS may suffer from the elevated risk of AF due to long-time NSAID therapy.

### The implications of Mendelian randomization and linkage disequilibrium regression score findings

Whilst our MR findings provide no evidence for the causal effect of genetic liability to AS on lifelong risk of AF, increased risk of AF in patients of AS is a non-neglectable problem in the real world. It might be more likely attributed to other factors such as traditional CV risk factors, inflammatory factors, and medications. According to the European League Against Rheumatism (EULAR) recommendations for CV risk management (2016 update) in patients with inflammatory joint disorders (Agca et al., 2017), our findings have strengthened the importance of interventions for prevention of AF in patients with AS, including the modification of traditional risk factors, the control of inflammation burden and the caution for NSAIDs usage.

Our LDSC findings support no evidence for genetic correlation between AS and AF at a genome-wide level. Interestingly, we discovered that the *IL-6R* SNP rs4129267 that was a pleiotropic SNP removed from our MR analysis has been previously related to CRP level, risk of AS, and risk of AF at genome-wide significance (International Genetics of Ankylosing Spondylitis Consortium et al., 2013). Another proteome-wide association study has found that the serum level of the soluble IL-6 receptor is affected by the genotype of rs4129267. Given the causal role of IL-6 level in AF, IL-6 might be a potential target and biomarker for prevention of AF in AS population, which requires clinical trials to validate. Furthermore, we postulate that despite insufficient evidence for a causal relationship, genetic colocalization at specific loci such as *IL-6R* or genetic correlation at local genome regions which might explain part of observational association between AS and AF through inflammatory pathways. Local genetic correlation analysis and colocalization analysis might be alternative approaches to explore the complex relationship between AS and AF.

### Strengths

This study has some strengths. First, the MR method avoided inherent biases in observation studies: confounding factors, reverse causation, and regression dilution. Second, large-scale GWAS datasets made statistic power more sufficient to detect the causality.

### Limitations

The study was subject to several limitations. First, this MR analysis was conducted within the European population; hence, the results cannot be well-extrapolated to the non-European population. Second, the GWAS summary statistic for AF did not provide the information of AF subtypes. Thus, we were unable to explore the causality of AS and any AF subtypes. Third, we cannot rule out potential genetic pleiotropy in this MR study. However, we excluded known SNPs related to AF and any confounder factors before our MR analysis. What’s more, pleiotropy tests were also indicative of no pleiotropy. Fourth, we did not perform a reverse MR analysis on the causation from AF to AS. However, it is biologically plausible that AF is not a cause of the incident AS due to the major genetic contribution of HLA-B27 to AS. Finally, this study only provides genetic evidence that there is no causation between AS and AF without involving environmental factors.

## Conclusion

The MR study provides no evidence of the causality between AS and AF. The mechanisms underlying the observational association between them remain to be further investigated. However, we cannot ignore the higher risk of AF in AS patients due to inflammation burden, traditional risk factors, and medications. Early management of AF is warranted for AS patients.

## Data Availability

The datasets used in this study can be available in the OpenGWAS database (https://gwas.mrcieu.ac.uk/datasets/) with the corresponding GWAS IDs (AS dataset: ebi–a–GCST005529; AF dataset: ebi–a–GCST006414).
